# Emergency science: Epistemological insights on the response to COVID-19 pandemics

**DOI:** 10.1017/ice.2020.209

**Published:** 2020-05-11

**Authors:** Carlos Magno Castelo Branco Fortaleza

**Affiliations:** 1 Department of Infectious Diseases, Botucatu School of Medicine, São Paulo State University (UNESP). City of Botucatu, São Paulo Sate, Brazil

*To the Editor*—In the year 2000, South Africa President Thado Mbeki was the spokesman of an organized movement of AIDS denialism, which still echoes in many countries, such as Russia.^1^ When facing pandemics, scientific denialism may adopt several faces and disguises. The “common cold” argument against the potential severity of COVID-19 spread through social media. The balance of socioeconomic versus sanitary achievements of lockdown may be contaminated by global ideological discussions and/or local political interests.^2^ Public health challenges become more intense when decisions depend on mathematical modeling and scarce, suboptimal empirical evidence. Such occurrences obviously have implications for infection control and patient safety.

When an ongoing pandemics require rapid translation of research findings into practice guidelines, the epistemological basis of modern public health must be discussed. Here, I submit the scientific basis of the response to the pandemic to critical scrutiny based on ideas from renowned philosophers of science. Additionally, I discuss the policy of disseminating knowledge in times of global emergency.

“Unlike (…) doctors, the scientist need not choose problems because they urgently need solution,” stated Thomas Kuhn (1922–1996) is his masterpiece *The Structure of Scientific Revolutions*.^3^ Kuhn was too young to have experienced 1918 influenza pandemics, but he did live the first decade of AIDS emergence. His statement that sciences develop inside or around a historically determined paradigm was highly praised by sociologists and anthropologists, and he influenced (nonorthodoxically, I must clarify) eminent epidemiologists.^4^ Different paradigms are incommensurable, an adjective by which Kuhn means that when scientist sees the world from different perspectives, their contradictions just cannot be solved by discussion or experiment. Well, we must ask if the germ theory is one of those Kuhnian paradigms. From Kuhn’s perspective, all the science raised in the pandemic response has a historical, but not epistemological, justification.

Karl Popper (1902–1994) dedicated all his life to explore what he called “the two fundamental problems in the theory of knowledge.” First is the problem of induction, as described by David Hume (1711–1776), which states that no matter how many observations (which we can translate to scientific evidence) of “A” followed by “B,” a causal association would always be a psychological rather than a logical conclusion. Second is the problem of demarcation, which is the need for a clear rule or boundary between what is scientific and what is not.^5^ Agreeing with Hume, Popper refused induction (the cornerstone of “evidence-based medice”) and proposed that scientists should be creative in imagining theories, then rigorous in testing them both rationally and empirically. Any theory will survive as the fittest while it resists empirically based refutations. Coherently, science is defined for its possibility of empiric falsification. How does this apply to scientists fighting COVID-19? From a Popperian perspective, public response would be strengthened by a network of mutually critical researchers. Although theoretical discussion and criticism cannot be paralyzing or move too slowly while we count the dead, the scientific community may be prepared to endorse changes in public policies whenever a theory is refuted and studies point to novel, more adequate strategies.

Of greater concern is Paul Feyrabend’s (1924–1994) “anything goes” statement in his famous book *Against Method*,^6^ or his criticism on modern medicine achievements in his later works. In a similar direction, New York University Philosopher Peter Unger’s (born 1942) skeptical argument that “nothing can ever be really known” is, in the best hypothesis, useless, and in the worst scenario, highly dangerous if spread over all public opinion.^7^

Finally, an interesting way out of the epistemological puzzle is provided by Imre Lakatos’s (1922–1974) insights on “Programs of Scientific Investigation.”^8^ Lakatos attempted to reconcile Popper’s and Kuhn’s ideas, and he had a productive dialogue with Feyerabend. Briefly, Lakatos imagined networks of mutual criticism (like Popper) in permanent rivalry. Occasionally, one of those programs gains advantage (becomes “progressive”) over others (which become “regressive”). He admits (like Kuhn) some historicity in the balance between them. Still, he fiercely advocates for a demarcation criterion for science. We must therefore assume that virological–epidemiological programs are progressive and should not only be heard by government officials but also must be given more expression in scientific journals.

This brings us to the final discussion. Peer-review has provided the basis for continuous Popperian–Lakatosian criticism, and at least in theory it provides a scientific quality badge to information. Both peer review and editing processes take time, which we do not have in the current pandemics. Hundred of studies are either published in preprint repositories or submitted to fast-track peer review.^9^ This obviously means loosening the critical parameters, a choice of speed over rigor. That trend is totally in accordance with Lakato’s predicted privileges for progressive programs. However, it requires a permanent critical attitude from the readers and a constant state of alert in the scientific community.

In conclusion, the response to COVID-19 does not require consensus. Criticism is perhaps the most precious principle of scientific thinking and practice. By submitting the role of science in responding to COVID-19 to the scrutiny of leading critics of mainstream science, we not only vaccinate our community against nihilistic arguments but also reinforce the human value of research activity. Research and scientific criticism must be exercised aiming to collaborate with public policies and avoiding messages of uncertainty and insecurity to the already sufficiently frightened population. Furthermore, even if we argue that there is no such thing as value-neutral science,^10^ we need to move our research away from political and corporate interests. Thus, in an era of extremism, science can rise as the pillar of democracy and as a movement to protect life.

## References

[1] Kalichman SC . Pence, Putin, Mbeki and their HIV/AIDS-related crimes against humanity: call for social justice and behavioral science advocacy. AIDS Behav 2017;21:963–967. 28130629 10.1007/s10461-017-1695-8

[2] Editorial. COVID-19: learning from experience. Lancet 2020;395:1011. 32222181 10.1016/S0140-6736(20)30686-3 PMC7194650

[3] Kuhn TS . The Structure of Scientific Revolutions. Chicago: University of Chicago Press; 1962.

[4] Susser M , Susser E . Choosing a future for epidemiology: I. Eras and paradigms. Am J Public Health 1996;86:668–673. 8629717 10.2105/ajph.86.5.668 PMC1380474

[5] Popper K . The Two Fundamental Problems in the Theory of Knowledge. London: Routledge; 2009.

[6] Feyerabend P . Against Method. London: New Left Books; 1975.

[7] Unger P . Ignorance: A Case for Scepticism. Oxford: Oxford University Press; 1975.

[8] Lakatos I . The Methodology of Scientific Research Programmes: Volume 1: Philosophical Papers. Cambridge: Cambridge University Press; 1980.

[9] Flier JS . Covid-19 is reshaping the world of bioscience publishing. Statnews website. https://www.statnews.com/2020/03/23/bioscience-publishing-reshaped-covid-19/, 2020. Published March 23, 2020. Accessed May 5, 2020.

[10] Lekka-Kowalik A . Why science cannot be value-free: understanding the rationality and responsibility of science. Sci Eng Ethics 2010;16:33–41. 19343542 10.1007/s11948-009-9128-3

